# *Actinobaculum schaalii* an emerging pediatric pathogen?

**DOI:** 10.1186/1471-2334-12-201

**Published:** 2012-08-28

**Authors:** Petra Zimmermann, Livia Berlinger, Benjamin Liniger, Sebastian Grunt, Philipp Agyeman, Nicole Ritz

**Affiliations:** 1 Department of Paediatrics, University Children’s Hospital, Berne, Switzerland; 2 Institute for Infectious Diseases, University of Berne, Berne, Switzerland; 3 Bioanalytica AG, Lucerne, Switzerland; 4 Department of Paediatric Surgery, University Children's Hospital, Berne, Switzerland; 5 Department of Neuropediatrics, Development and Rehabilitation, University Children’s Hospital, Berne, Switzerland; 6 Infectious Diseases Unit, University Children’s Hospital Berne, Berne, Switzerland; 7 Infectious Diseases Unit, University Children’s Hospital Basel, Spitalstrasse 33, Basel, CH-4031, Switzerland

**Keywords:** *Actinobaculum schaalii*, Children, Emerging infection, Urinary tract infection, Gram-positive, Antimicrobial susceptibility

## Abstract

**Background:**

*Actinobaculum schaalii* was first described as a causative agent for human infection in 1997. Since then it has mainly been reported causing urinary tract infections (UTI) in elderly individuals with underlying urological diseases. Isolation and identification is challenging and often needs molecular techniques. *A. schaalii* is increasingly reported as a cause of infection in humans, however data in children is very limited.

**Case presentation:**

We present the case of an 8-month-old Caucasian boy suffering from myelomeningocele and neurogenic bladder who presented with a UTI. An ultrasound of the urinary tract was unremarkable. Urinalysis and microscopy showed an elevated leukocyte esterase test, pyuria and a high number of bacteria. Empiric treatment with oral co-trimoxazole was started.

Growth of small colonies of Gram-positive rods was observed after 48 h. Sequencing of the 16S rRNA gene confirmed an *A. schaalii* infection 9 days later. Treatment was changed to oral amoxicillin for 14 days. On follow-up urinalysis was normal and urine cultures were negative.

**Conclusions:**

*A.schaalii* is an emerging pathogen in adults and children. Colonization and subsequent infection seem to be influenced by the age of the patient. In young children with high suspicion of UTI who use diapers or in children who have known abnormalities of their urogenital tract, infection with *A. schaalii* should be considered and empiric antimicrobial therapy chosen accordingly.

## Background

The genus *Actinobaculum* was first described as a cause of human infection in 1997 [1]. Since the initial description of the genus *A. schaalii* has been the most frequently reported human pathogen. It has mainly been reported causing urinary tract infection (UTI) in elderly people with underlying urological diseases. Like other *Actinomyces-like* organisms it is suspected to be part of the commensal flora of the human urogenital tract [1]. Since the introduction of molecular techniques into routine microbiological diagnosis, *A. schaalii* has increasingly been reported as a cause of infection in humans [2,3]. However data in children with *A. schaalii* infection is very limited [4].

## Case presentation

An 8-month-old boy with a lumbosacral myelomeningocele surgically repaired shortly after birth and a hydrocephalus due to an Arnold-Chiari malformation was seen at our clinic for a routine appointment. He had not previously suffered from a UTI and did not need regular catheterization. On presentation he was afebrile and his physical examination was normal. A cystomanometry performed on the same day revealed an elevated bladder pressure and a residual urine volume of 25 ml, confirming the expected neurogenic bladder dysfunction. An ultrasound of the urinary tract was unremarkable. Urinalysis from a catheter specimen showed an elevated leukocyte esterase activity Microscopy of the urine showed more than 20 leukocytes, 5 to 10 erythrocytes, plus a high number of bacteria per high-power field. A UTI was postulated and the patient was empirically treated with oral co-trimoxazole (trimethoprim/sulfamethoxazole 5/25 mg/kg twice daily) for 7 days. According to standard procedure urine cultures were set up on a CHROMagar Orientation plate (Becton Dickinson AG, Basel, Switzerland) and a Columbia-Colistin-Nalidixic acid agar (CNA) plate (Becton Dickinson, Basel, Switzerland) with 5% sheep blood. Growth of small colonies was observed on the CNA plate after 48 h of incubation at 35°C with 5% CO_2_, corresponding to 10^4^ colony forming units (CFU)/ml. The growing Gram-positive rods were catalase-negative and could not be identified by the API Coryne System (bioMérieux, Geneva, Switzerland). For further identification sequencing of the 16S rRNA gene by MicroSeq500 16S rDNA bacterial identification kit (Applied Biosystems, Darmstadt, Germany) was performed. Nine days later Basic Local Alignment Search Tool (BLAST) analysis of the resulting 477 base pair sequence was available and showed 100% identity with *A. schaalii* strain BD04-00146 (GenBank accession number AY957507.2).

Two weeks after the initial appointment the patient was reviewed. Microscopy of the urine continued to show 10 to 20 leukocytes and 10 to 20 erythrocytes per high-power field with presence of bacteria. Blood analysis showed a C-reactive protein of 9 mg/l and a leukocyte count of 7 × 10^9^/l. The combination of a repeatedly abnormal urinalysis and growth of *A. schaalii* in monoculture was considered diagnostic for a UTI. According to current recommendations for the treatment for *A. schaalii* infection oral amoxicillin (15 mg/kg three times daily) was started [3,5]. The urine culture from the same day showed growth of coagulase-negative staphylococci. Eight days after starting amoxicillin, results of the urinalysis were normal and urine cultures were negative. The treatment with amoxicillin was continued for a total duration of 14 days.

## Discussion

In children UTI is usually caused by gram-negative organisms, including *Escherichia coli, Klebsiella* spp.*, Pseudomonas* spp. and *Proteus* spp.[6]. *A. schaalii*, a recently described pathogen, has to date mainly been associated with UTI in elderly individuals.

A total of 47 cases of *A. schaalii* infection have been published to date [1,3,4,7-20]. Of these, 41 cases (87%) were reported in adults and only six cases in children under the age of 15 years (Table 1). All of those six children were symptomatic and treated with antibiotics. Two of the three children with cystitis and the child with pyelonephritis were febrile. In the case of the 15-year old boy who presented with cystitis and the girl with an intradural abscess the temperature was not specified in the case report. Our case is, to our knowledge, the youngest child currently described with an *A. schaalii* UTI. It is also the first case where *A. schaalii* was treated in an asymptomatic child. In previous case reports from both children and adults a high proportion of individuals with an *A. schaalii* UTIs had an underlying urogenital pathology (83% and 85%, respectively). All of the reported children were either using diapers or had enuresis. In two children a second pathogen was isolated: *Klebsiella pneumoniae* in a 3-year-old girl with recurrent UTI [21] and non-hemolytic streptococci in a 9-month-old girl with intradural abscess [10]. Further clinical details of all pediatric cases are summarized in the Table 1. 

**Table 1:** **Clinical details of reviewed pediatric cases with* Actinobaculum schaalii *infection**

**Age (years)**	**Sex**	**Clinical presentation**	**Specimen with*A. schaalii***	**Concomitant flora**	**Underlying urogenital tract pathologies**	**Other underlying risk factors**	**Antibiotic treatment**	**Duration of treatment(days)**	**Outcome**	**Reference**
0.66	m	cystitis	urine	none	neurogenic bladder	myelomeningocele diaper	co-trimoxazole followed by amoxicillin	7 14	recovery	our case
0.75	f	cauda equina syndrome	pus from intradural abscess	*non-hemolytic streptococci*	none	syringomyelia diaper	penicillin, metronidazol	not specified	recovery	10
3	f	cystitis	urine	*Klebsiella pneumoniae*	none	recurrent UTI diaper	trimethoprim followed by amoxicillin	7 10	recovery	21
5	m	pyelonephritis	urine	none	pyeloureteral junction obstruction	inborn right hemispheric tissue lesions with left-sided hemiplegia epilepsy	amoxicillin/clavulanic acid followed by vancomycin	2 14	recovery	4
13	m	cystitis	urine	none	neurogenic bladder	enuresis	pivampicillin followed by mecillinam followed by pivampicillin	20 10 14	re-infection after 1 year recovery	21
15	m	cystitis	urine	none	neurogenic bladder vesicoureteral reflux bladder diverticulum	myelomeningocele paraplegia	amoxicillin/clavulanic acid	7	recovery	*

*Personal communication, Reto Lienhard, Department of Microbiology, La Chaux-de-Fonds.

Our case highlights the clinical features and challenges in diagnosis and management of *A. schaalii* UTI. Isolation and identification of *A. schaalii* using standard laboratory techniques is difficult. Notably, *A. schaalii* requires CO2 enriched culture conditions and subsequent identification requires 16S rRNA sequencing. Therefore the presence of Gram-positive rods in microscopy with a negative culture result should prompt the search for unusual pathogens including *A. schaalii.*

This may lead to a significant delay in identification resulting in prolonged or repeated antimicrobial therapy. Its resemblance to normal skin flora further impedes correct identification of the pathogen. In addition slow growing bacteria such as *A. schaalii* can get overgrown by faster-growing bacteria and therefore remain undetected. For this reason we opted to continue treatment in our patient until urinalysis had normalized. Implementation of the newer Matrix-assisted laser desorption/ionization-Time Of Flight (MALDI-TOF) technology in routine diagnostic procedures may allow more rapid identification of *A. schaalii* in future, which might change the frequency of its detection.

*A. schaalii* has traditionally been thought to be part of the commensal flora of the human urogenital tract [1]. However, there is evidence that colonization with *A. schaalii* is dependent on risk factors including age, the presence of diapers and enuresis. For example, in asymptomatic patients above 60 years of age with negative results in a urine dipstick test, a real-time quantitative PCR assay detected *A. schaalii* in 22% of urine samples [2]. Similarly, in children suspected for UTI and negative results in urine dipstick test from midstream urine samples, PCR for *A. schaalii* from urine was positive in 36% (5 of 14) of children below three years of age but negative in all 15 children tested between 3 and 15 years of age [21].

Colonization seems to be a risk factor for infection with *A. schaalii* in children and adults as most case reports have been described in the same age group where colonization most frequently occurs.

In our patient urine culture grew 10^4^ CFU/ml *A. schaalii* and in contrast to previous reports in children with *A. schaalii* UTI there were no clinical symptoms [4,10,21]. Similar colony counts have been found in children considered to be colonized with *A. schaalii*, however, none of these children had concomitant positive leukocyte esterase tests or pyuria [21]. In our patient the combination of a repeatedly pathologic urinalysis including a positive leukocyte esterase test and pyuria together with growth of 10^4^ CFU/ml of a recognized uropathogen in monoculture was considered sufficient evidence for the diagnosis of a cystitis. The vast majority of *A. schaalii* isolates are resistant to co-trimoxazole and ciprofloxacin, which are frequently used as empiric treatment in UTI in children and adults respectively. Current recommendations for the choice of antibiotic treatment for *A. schaalii* infection are amoxicillin or cephalosporins [5]. There is limited data from *in vitro* susceptibility testing suggesting that gentamicin, vancomycin, linezolid, nitrofurantoin and mecillinam are potential alternative treatment options [5,22]. The optimal duration of antibiotic treatment for *A. schaalii* infection is currently unknown. Most case reports suggest treatment duration of 7 to 14 days. Therefore we repeated the urine culture after eight days of antibiotic treatment, and stopped treatment after 14 days when negative culture results were available.

## Conclusions

*A. schaalii* is an emerging pathogen in both children and adults. Colonization and subsequent infection seem to be influenced by a number of factors in particular age of the patient. In young children with high suspicion of UTI who use diapers or children who have known abnormalities of their urogenital tract, infection with *A. schaalii* should be considered and empiric antimicrobial therapy chosen accordingly. Further studies are needed to evaluate additional risk factors and to define optimal choice and duration of antibiotic treatment.

## Consent

Written informed consent was obtained from the patient’s parents for publication of this case report. A copy of the written consent is available for review by the Series Editor of this journal.

## Competing interests

The authors declare that they have no competing interests.

## Authors’ contributions

PZ wrote the draft of the manuscript and preformed the literature review. BL and SG have been involved in patient’s clinical care. LB preformed the microbiological and molecular diagnoses and wrote of the relevant section in the case report. PA and NR reviewed the manuscript. All authors read and approved the final manuscript.

## Authors’ information

Philipp Agyeman and Nicole Ritz joint authorship.

## Pre-publication history

The pre-publication history for this paper can be accessed here:

http://www.biomedcentral.com/1471-2334/12/201/prepub
